# RNA variant identification discrepancy among splice-aware alignment algorithms

**DOI:** 10.1371/journal.pone.0201822

**Published:** 2018-08-02

**Authors:** Ji Hyung Hong, Yoon Ho Ko, Keunsoo Kang

**Affiliations:** 1 Division of Oncology, Department of Internal Medicine, College of Medicine, The Catholic University of Korea, Seoul, Republic of Korea; 2 Cancer Research Institute, College of Medicine, The Catholic University of Korea, Seoul, Republic of Korea; 3 Department of Microbiology, College of Natural Sciences, Dankook University, Cheonan, Republic of Korea; CNRS UMR7622 & University Paris 6 Pierre-et-Marie-Curie, FRANCE

## Abstract

Next-generation sequencing (NGS) techniques have been generating various molecular maps, including transcriptomes via RNA-seq. Although the primary purpose of RNA-seq is to quantify the expression level of known genes, RNA variants are also identifiable. However, care must be taken to account for RNA’s dynamic nature. In this study, we evaluated the following popular splice-aware alignment algorithms in the context of RNA variant-calling analysis: HISAT2, STAR, STAR (two-pass mode), Subread, and Subjunc. For this, we performed RNA-seq with ten pieces of invasive ductal carcinoma from breast tissue and three pieces of adjacent normal tissue from a single patient. These RNA-seq data were used to evaluate the performance of splice-aware aligners. Surprisingly, the number of common potential RNA editing sites (pRESs) identified by all alignment algorithms was less than 2% of the total. The main cause of this difference was the mapped reads on the splice junctions. In addition, the RNA quality significantly affected the outcome. Therefore, researchers must consider these experimental and bioinformatic features during RNA variant analysis. Further investigations of common pRESs discovered that BDH1, CCDC137, and TBC1D10A transcripts contained a single non-synonymous RNA variant that was unique to breast cancer tissue compared to adjacent normal tissue; thus, further clinical validation is required.

## Introduction

RNA editing is a post-transcriptional process mediated by specific enzymes that induce changes in RNA sequences compared to the original DNA sequence [1]. It was originally reported in trypanosome mitochondria [2], and later found in many eukaryotic and prokaryotic species [3, 4]. The main type of RNA editing in mammals is A-to-I editing, which converts adenosine to inosine; this process is catalyzed by a family of adenosine deaminases that act on double-stranded RNA (ADARs) [5]. Most A-to-I editing events in humans occur in non-coding regions, prevalently in Alu elements, which are the human genome’s most abundant retrotransposons [6, 7]. In addition to A-to-I editing, C-to-U editing, which converts cytidine to uridine on both RNA and DNA, has been characterized in higher eukaryotes [8, 9]. A recent study [10] that analyzed 8,551 human samples from 552 individuals revealed that a large number of A-to-I RNA edits occur dynamically in various tissues in a spatiotemporal manner. Since the RNA-editing process can alter the amino acid sequence of an encoded protein, the clinical relevance of RNA-editing sites (RESs) has recently been recognized [11, 12].

Recent advances in DNA sequencing technology that have been led by next-generation sequencing have opened up a new era of genomics by providing a vast number of genome-wide molecular maps regarding genomes, transcriptomes, and epigenomes [13–15]. Recently, the identification of RESs via NGS has been investigated in many model organisms. Although NGS can accurately identify genome-wide DNA (or RNA) variants at a single-base resolution, many false positive detections have been reported [16]. Compared with DNA variant analysis, RNA variant analysis is considered susceptible to false positives due to the dynamic nature of RNAs [17]. As a messenger conveying protein-coding information from DNA to proteins, RESs can directly alter amino acid changes, which can be missed by the DNA-based approach. If RNA editing is a driver for the onset (or progression) of diseases such as cancer, the accurate identification of RESs can dramatically improve our knowledge, which is currently limited to DNA variants. Therefore, a reliable analysis scheme that can detect RESs using RNA-seq is needed. However, there are no thorough evaluations of RNA-editing analysis pipelines, and RNA-editing analysis is more complex than DNA variant analysis due to the dynamic changes of RNA levels between samples, even in biological replicates. Computationally, RESs are typically identified through a five-step process: i) trimming low-quality portions of sequenced reads, ii) aligning trimmed reads to the reference genome, iii) calling RNA variants, iv) annotating RNA variants based on gene annotations, and v) extracting RNA variants (so-called RESs) that do not overlap with DNA variants. Among these steps, the alignment step is the most critical, since the subsequent remaining steps are based on reads mapped to the genome. Thus, we mainly focused on evaluating the alignment step in a typical RNA variant analysis scheme in this study. We selected the following popular and fast alignment tools for evaluations: STAR, HISAT2, Subjunc, and Subread. Since there are no true RNA-editing sites set for evaluation, we performed RNA-seq with one invasive ductal carcinoma tissue and its adjacent normal tissue biopsied from a patient with breast cancer. The cancer and normal tissues were physically divided into ten and three pieces respectively and then analyzed using RNA-seq. Our evaluation revealed that the quality of RNAs and the selection of alignment tools substantially affected the identification of RNA variants, including RESs. Thus, researchers should be aware of these factors when identifying RNA variants using RNA-seq.

## Materials and methods

### RNA isolation and RNA-seq

One tissue of invasive ductal carcinoma (luminal B subtype) from breast tissue and a corresponding adjacent normal tissue were biopsied from a single Korean woman with informed consent. Then, we cut the tumor and adjacent normal tissues into ten and three pieces, respectively. Poly(A) RNA was purified from 1 g total RNA from each sample, and cDNA was synthesized using SuperScript II (Invitrogen). Sequencing libraries were prepared using the TruSeq RNA library preparation kit (Illumina) and sequenced using HiSeq 2500 (Illumina). The RNA-seq data was deposited in GEO under accession number (GSE110114). The study was approved by the institutional review board of Catholic Medical Center (approval no. UC17TISI0015). The patient signed an informed consent form before study-related procedures were conducted.

### Genome-wide RNA variant calling

Low-quality portions of paired-end reads were trimmed using Trim galore (version 0.4.2; https://www.bioinformatics.babraham.ac.uk/projects/trim_galore/) with cutadapt (version 1.1.2) [18]. Sequenced reads were mapped to two reference human genomes (hg38 and KOREF1.0 assemblies) using the following alignment tools with default parameters: Subread or Subjunc (version 1.5.1) [19]; HITSAT2 (version 2.0.5) [20]; and STAR or STAR2 (two-pass mode) (version 2.5.2b) [21]. The PCR duplicate removal of mapped reads was conducted using Sambamba (version 0.6.5) for a duplicate-removed (RmDup) set [22]. The mapped reads of RNA-seq samples in each group (breast cancer or normal) were also merged to evaluate RNA-seq variant-calling using Sambamba. Strelka2 (version 2.8.3) was used to identify RNA variants from the mapped reads with the option for RNA-seq (—rna) [23]. Finally, RNA variants were selected that passed the following filters: annotated as “PASS”, genotype quality (GQ) > 15, and read depth (DP) > 10. The RNA variants were annotated with known genes’ and single nucleotide polymorphisms’ (SNPs) information using ANNOVAR (version 2017JUL16) [24]. pRESs were defined as the RNA variants that did not overlap with known SNPs.

### RNA quality check with RNA-seq data

The quality of RNAs in each sample was assessed using RSeQC (version 2.6.4; tin.py) [25] with each mapped file (.bam). The transcript integrity number (TIN) was used to evaluate the RNA integrity at the transcript level.

### Quantification of gene expression

The abundances of known genes (GENCODE comprehensive gene annotation, version 27; https://www.gencodegenes.org/releases/current.html) were estimated using Cufflink (version 2.2.1) [26].

## Results

### Comparison of alignment tools for RNA variant calling analysis

In this study, “RNA variant” is defined as a single nucleotide variant observed in RNA-seq data, while “potential RNA editing site (pRES)” is defined as an RNA variant that did not overlap with the known 325,083,445 DNA variants.

**Fig 1:**
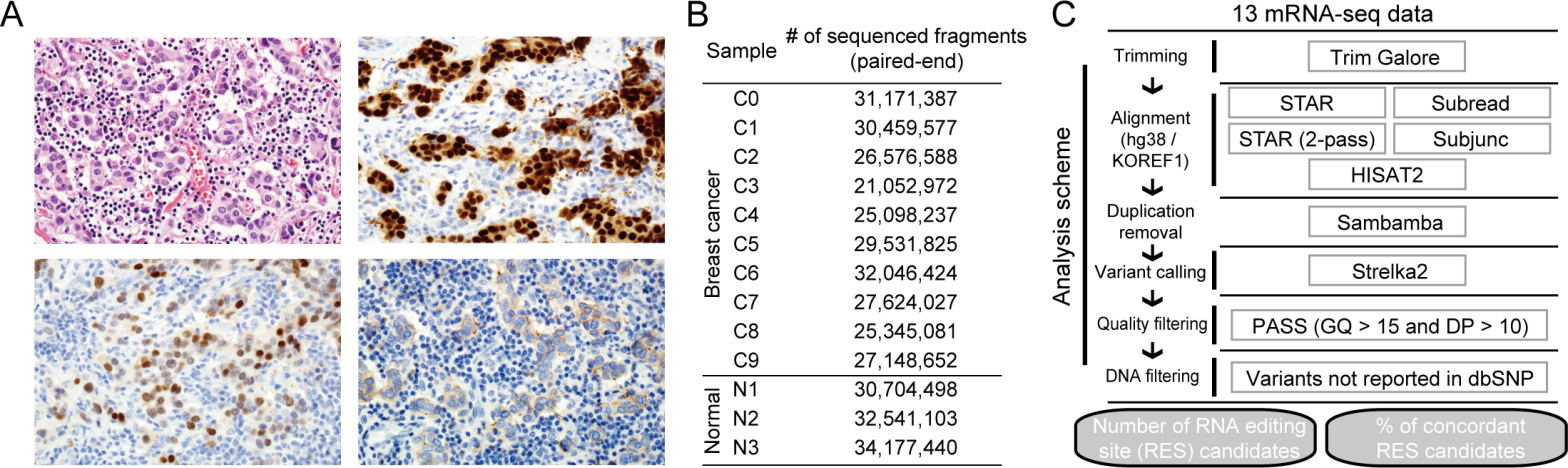
Overview of the evaluation strategy. (A) H&E (top left), ER (top right), PR (bottom left), and HER2 (bottom right) staining of invasive breast cancer tissues biopsied from a single patient (luminal B subtype). H&E x20, ER (IHC x40), PR (IHC x40), and HER2 (IHC x40). (B) The number of sequenced fragments that were used for alignment was counted. (C) A depiction of the RNA variant analysis scheme used in this study.

Unlike DNA variants, the identification of RNA variants can be affected by several factors due to RNA’s dynamic and unstable nature. Due to the lack of a gold-standard RNA-seq set for evaluation, we performed RNA-seq with ten pieces (C0–C9) of invasive ductal carcinoma tissue and three pieces (N1–N3) of adjacent normal tissue from a single Korean breast cancer patient (luminal B subtype) (Fig 1A). The cancer and adjacent normal tissues were physically divided into pieces by the same person immediately post-biopsy. Our evaluation was based on the following assumption: if an RNA variant is true, then the RNA variant should be identified in all ten tumor pieces (or three normal pieces) using RNA-seq. For this, paired-end (100bp) unstranded RNA-seq was performed by a company (Theragen, South Korea). The average number of sequenced RNA fragments was 28,729,062, which meant that there were approximately 5,745,812,477 bp of RNAs sequenced per sample (Fig 1B).

**Fig 2:**
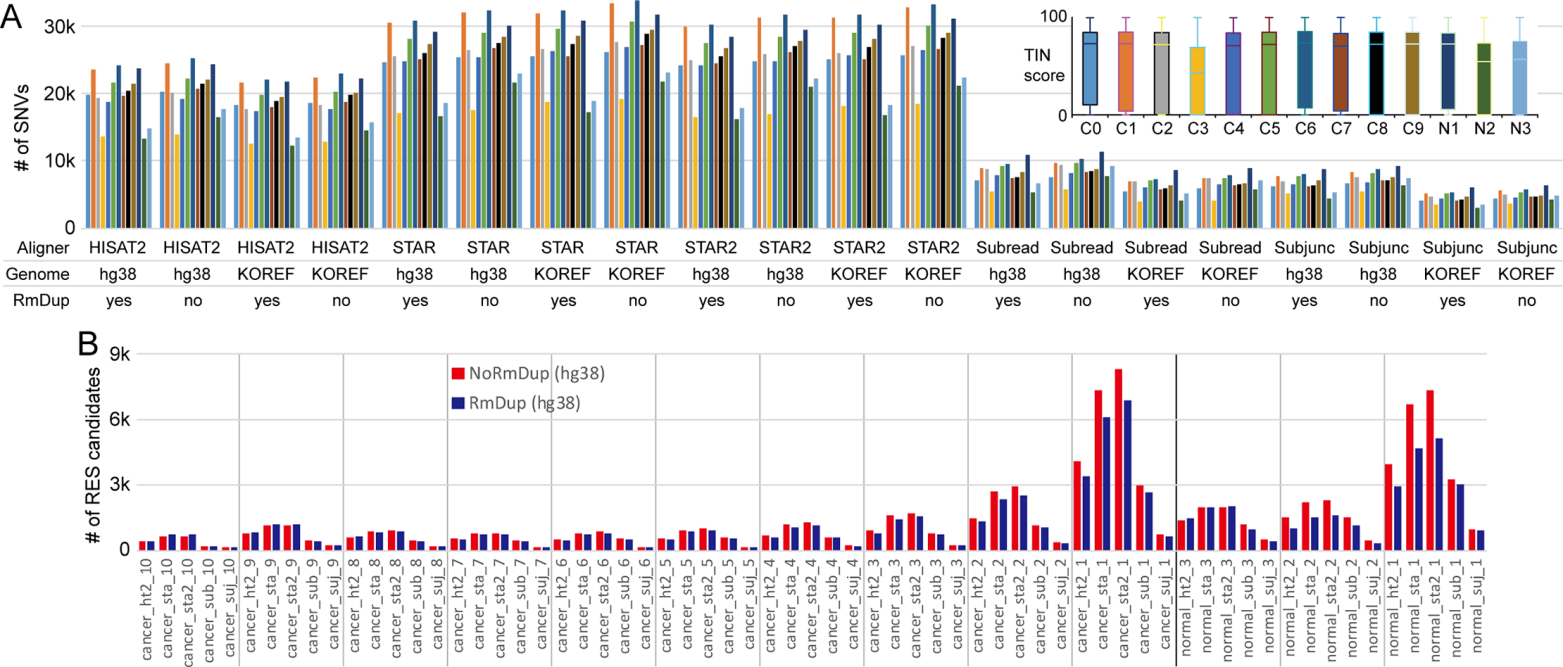
Comparison of alignment methods for RNA variant analysis. (A) The number of RNA variants (single nucleotide variants; SNVs) in each sample (C0–C9 or N1–N3) was calculated. The quality of RNAs was measured by means of transcript integrity number (TIN) score (upper right). (B) The number of pRESs was counted according to the group defined by the number of samples for which pRESs were detected. RmDup, duplication removal; ht2, HISAT2; sta, STAR; sta2, STAR (two-pass mode); sub, Subread; suj, Subjunc.

Several analysis steps should be considered for accurately identifying RNA variants. We followed an analysis scheme in this study (Fig 1C). We attempted to evaluate thoroughly one of the most critical steps, the alignment step. There are typically three things to be decided when conducting the alignment step: selecting an alignment tool, choosing a reference genome, and whether to remove PCR-duplicates after the alignment (i.e., duplicate removal). First, we determined how many RNA variants could be identified and how many of these could concordantly be identified between samples within a given group (cancer or normal), depending on the combination of the selection. In this analysis, we did not exclude any DNA variants, and thus most RNA variants might reflect DNA variants. Our result indicates that the selection of alignment tools was the most important factor that predominantly affected the number of identified RNA variants (Fig 2A). For example, HISAT2 identified 24,546 RNA variants in the C1 sample, while STAR (two-pass mode) detected 32,737 RNA variants when reads were mapped to the human reference genome (KOREF1.0 assembly) without duplicate removal. Intriguingly, Subjunc detected the fewest RNA variants and STAR identified the most RNA variants across all samples. Unlike the difference that depended on the alignment tools, the number of identified RNA variants was less affected by the other factors such as the choice of genomes for alignment or the use of duplicate removal (Fig 2A). We focused on potential RNA editing sites (pRESs) by filtering out DNA variants from the identified RNA variants using the 325,083,445 DNA variant information from the dbSNP database (build 150). Next, we evaluated which alignment methods concordantly identified as pRESs between samples within a given group (cancer or normal). For this, we counted the number of samples on each pRES (Fig 2B); this analysis showed that STAR outperformed the other methods in all cases. However, this did not mean that STAR accurately identified pRESs, but rather that it concordantly identified as many pRESs as possible. Although an expert performed all the experiments at the same time, RNAs from one sample (C3) of the cancer tissue and two samples (N2 and N3) of the normal tissue seemed to be low- and moderate-quality, respectively, as determined by the transcript integrity number (TIN) score. The average of median TIN values (where higher is better) in all samples was 76.1, while the C3 sample showed a median TIN value of 59.6, indicating low-quality RNAs (Fig 2A). The RNA quality and the number of identified RNA variants seemed positively correlated. For example, the number of RNA variants was small in C3, N2, and N3 samples regardless of alignment methods (Fig 2A). Therefore, researchers should be aware of these characteristics when performing RNA variant analysis.

### Performance of RNA alignment algorithms

**Fig 3:**
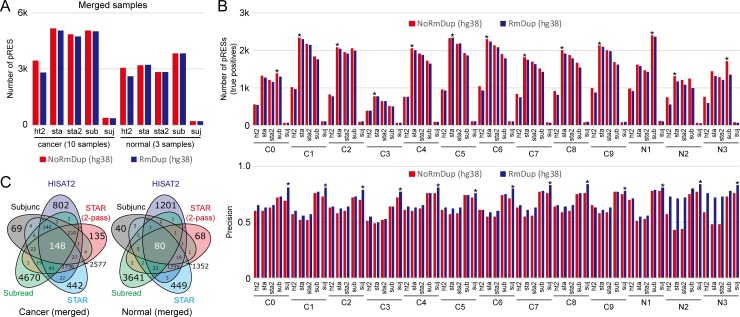
Performance evaluation of the alignment methods. (A) The number of pRESs identified by each aligner with merged cancer or normal RNA-seq data was counted. (B) The number of pRESs that coincided with those identified in each individual sample was counted (top). Precision was defined as the number of overlapped pRESs between merged RNA-seq and individual RNA-seq divided by the total number of pRESs identified in the given individual RNA-seq data (bottom). (C) The Venn diagrams show the number of overlapped pRESs identified by the five alignment tools.

The performance of alignment methods was assessed by independently merging ten cancer and three normal RNA-seq reads that were mapped by each alignment tool into cancer and normal merged sets, respectively. pRESs identified in each merged set were defined as a true set and used to calculate the precision and recall of each individual sample (C0–C9 or N1–N3) for a given alignment method. The read depth (DP) cutoffs for pRES calling in cancer and normal groups were scaled up to 100 and 30, respectively, according to the number of samples merged. The results showed that STAR and Subread identified the largest numbers of pRESs (Fig 3A) and that Subjunc detected the fewest pRESs in all cases, similar to the previous result (Fig 2A). This tendency was also observed when the number of true positives (Fig 3B) was calculated. However, regarding precision, Subjunc was highest, followed by Subread, and then HISAT2 (Fig 3B). Duplication removal slightly increased precision in most cases. In contrast, STAR showed the highest recall, followed by Subread in most cases (S1 Fig). However, these results did not indicate that any alignment tool outperformed the others, since each true set was defined as the identified pRESs when using the same alignment method. Next, we attempted to identify the pRESs detected by all five alignment methods (with duplicate removal applied) that were concordantly identified in the cancer or normal merged RNA-seq data. Surprisingly, very few common pRESs were detected: 148 out of 10,847 pRESs in cancer and 80 out of 8,234 pRESs in normal merged data (Fig 3C). This result indicated that the identification of RNA variants including pRESs was highly dependent on the alignment algorithm, highlighting the importance of alignment tool selection in RNA variant analysis.

### The discrepancy between alignment algorithms

**Fig 4:**
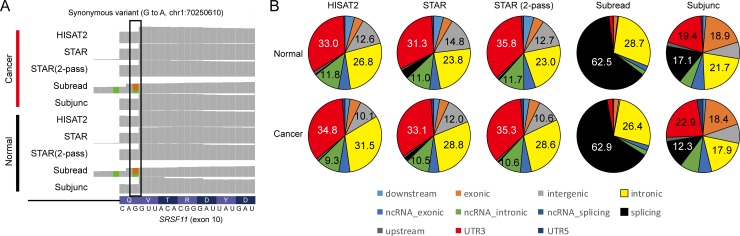
Comparison of the five alignment tools. (A) Example of a discordant RNA variant located in the 10th exon of the *SRSF11* gene. (B) Pie charts show the proportions of pRESs according to coding and noncoding (nc) gene-related features.

Since very few pRESs were commonly identified by all five aligners, we manually examined mapped reads to determine the cause of discrepancy via IGV [27]. Interestingly, Subread reported a pRES located at a splicing junction of the 10th exon in the *SRSF11* gene, but not the other aligners (Fig 4A). Therefore, we calculated the frequency of pRESs according to location based on various coding and noncoding (nc) gene-related features such as splicing junction, exon, intron, 5'-UTR (UTR5), and 3'-UTR (UTR3). Surprisingly, 62.5% (in normal) and 62.9% (in cancer) of pRESs identified by Subread were located on splicing junctions, while HISAT2, STAR, and STAR (two-pass) detected < 5% of pRESs on splice junctions (Fig 4B). In general, pRESs identified by HISAT2, STAR, and STAR (two-pass) were found in similar proportions on the coding and noncoding gene-related locations. To further confirm this tendency, we reanalyzed six RNA-seq data from a human breast cancer study (GSE75688) including single-cell RNA-seq data [28] and eight RNA-seq data from a mouse study (GSE79477). The results showed that STAR identified the largest number of pRESs, while Subjunc detected the fewest pRESs in all human RNA-seq samples examined whether pooled, tissue-based, or single-cell RNA-seq (S2A Fig). The same tendency was also observed in eight mouse RNA-seq samples (S2C Fig). Thus, the observed discrepancy between alignment tools seemed to be due to aligners’ algorithmic differences. In addition, we observed that many pRESs detected by Subread were located in splicing junctions, unlike the others (S2B Fig), which is similar to the previous result (Fig 4B). Therefore, these characteristics should be investigated thoroughly to ensure the correct identification of RESs for RNA-editing analysis.

### Genes with non-synonymous pRESs specific to breast cancer

**Fig 5:**
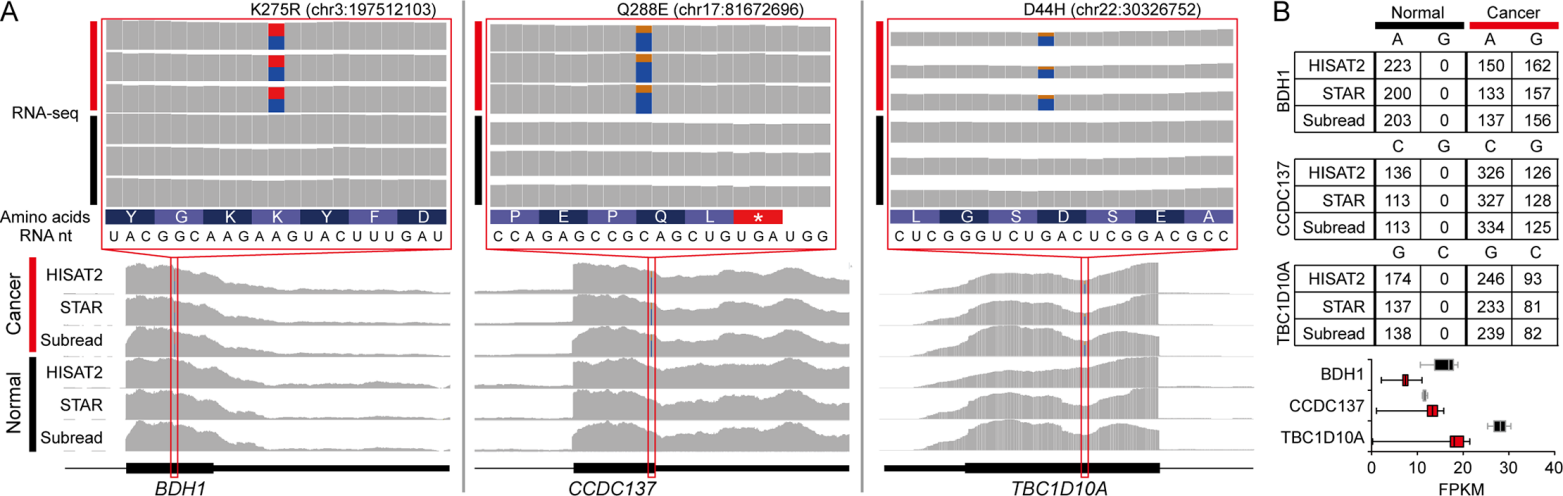
pRESs specific to breast cancer tissue. (A) Snapshots of mapped reads show pRESs and associated amino acid changes. (B) The number of mapped reads at the pRES sites was counted (top). The bar graphs show the expression levels of pRES-associated genes (bottom).

Potential driver pRESs that might be involved in the onset or progression of breast cancer were identified by manually investigating the 47 pRESs that were detected by all five alignment methods and also uniquely found in cancer tissue but not adjacent normal tissue (S1 Table). Among them, only six pRESs, which resided in the *IGHV-3*, *IGHV3-11*, *BDH1*, *CCDC137*, *TBC1D10A*, and *TRL10* genes, were non-synonymous variants that could alter the amino acid sequence of a given protein. Of those, the *IGHV-3* and *IGHV3-11* gene loci harbored many discordant RNA variants between aligners due to the complexity of these loci for mapping. In addition, one pRES present in the last exon of the *TRL10* gene could not be compared since the gene was weakly expressed in adjacent normal tissue (almost no reads were mapped at the pRES). Therefore, *BDH1*, *CCDC137*, and *TBC1D10A* were finally examined. Based on the pRESs, the following amino acid changes were predicted in cancer tissue compared to adjacent normal tissue: K275R in *BDH1*, Q288E in *CCDC137*, and D44H in *TBC1D10A* (Fig 5A). All mapped reads by all five aligners contained altered RNA nucleotides (Fig 5B), suggesting that these pRESs were not false positives. The expression levels of the *BDH1* and *TBC1D10A* genes were downregulated, while the *CCDC137* gene was upregulated in cancer tissue compared to adjacent normal tissue (Fig 5B). Further investigations of these pRESs will help identify genuine RNA editing sites that influence the onset or progression of breast cancer.

## Discussion

In this study, we mainly evaluated the alignment step, which is the most critical step for RNA variant analysis. It is reasonable to assume that RNA-seq alignment algorithms yield nearly identical results when using the same data. However, the identification of RNA editing sites through RNA-seq is extremely sensitive to alignment algorithms since this task is similar to finding a needle in a haystack. Therefore, performing a comprehensive evaluation of alignment algorithms for RNA editing analysis was the motivation of this evaluation study.

The present study performed RNA-seq using ten pieces of the same cancer tissue and three pieces of the same adjacent normal tissue biopsied from a patient with breast cancer. Since our evaluation of alignment algorithms was solely based on these RNA-seq data, our interpretation might be biased toward our RNA-seq dataset. Nevertheless, a series of evaluations discovered several important issues to take into account when studying RNA variants using RNA-seq. First, RNA quality significantly affects on outcome. In our case, one (C3) of the ten pieces from the cancer tissue seemed to be degraded even though an expert performed all experiments at the same time. The number of RNA variants was positively correlated with RNA quality, which was measured by the TIN score regardless of aligners (Figs 2A and 3B). Therefore, we recommend that RNA-seq data with a median TIN score of around 80.0 should be used for RNA editing analysis, although this score depends on sequencing types (e.g., single-cell RNA-seq, total RNA-seq, and mRNA-seq) as well as the gene set used for the TIN analysis. Second, the choice of alignment tools is an important consideration. Our evaluations indicated that 62% of pRESs identified by Subread were located at splice junctions, while HISAT2, STAR, and STAR (two-pass) showed similar distributions of pRESs (Fig 4). This algorithmic difference resulted in the inconsistent identification of pRESs between aligners (Fig 3C and S2B Fig). This characteristic is due to the purpose of the Subread program, which was developed to quickly quantify expression levels of whole genes without considering exon-exon junctions [19]. Therefore, Subread should only be used for the quantification of expression levels of genes but not for RNA-editing analysis. Although there were some discrepancies between aligners, we clearly identified the following genes with pRESs that can alter a single amino acid in a given protein unique to breast cancer tissue (Fig 5). The BDH1 protein, one of the rate-limiting enzymes that are required for ketone production, has been linked to breast cancer [29]. The TBC1D10A protein, a member of the GTPase-activation protein TBC1 domain family, is involved in exosome secretion by interacting with a Rab family member protein called Rab35 [30, 31]. The CCDC137 protein has been reported as a centromere-associated protein called cPERP-B [32]. Overall, our evaluations highlighted critical points that should be taken into account when identifying RNA variants using RNA-seq and discovered three novel pRESs that have not been reported in invasive ductal carcinoma studies.

## Supporting information

S1 Fig Performance evaluation of the alignment methods. Recall was defined as the number of overlapped pRESs between merged RNA-seq and individual RNA-seq divided by the total number of pRESs identified in the given merged RNA-seq data (bottom).
(TIF)

S2 Fig Comparison of the alignment methods with different RNA-seq data sets. (A) The bar graph shows the number of pRESs identified in the six samples from a human RNA-seq data set (GSE75688) [28]. (B) Pie charts show the proportions of pRESs according to coding and noncoding (nc) gene-related features. (C) The bar graph shows the number of single-nucleotide variants (SNVs; not filtered by known single nucleotide polymorphisms) identified in the eight samples from a mouse RNA-seq data set (GSE79447).
(TIF)

S1 Table The list of identified pRESs using merged cancer and normal RNA-seq. (XLSX)
