# Supplementary figures and images for: RNA variant identification discrepancy among splice-aware alignment algorithms
Source: PLoS One. 2018 Aug 2;13(8):e0201822. doi: 10.1371/journal.pone.0201822 (PMC6072070; doi:10.1371/journal.pone.0201822)

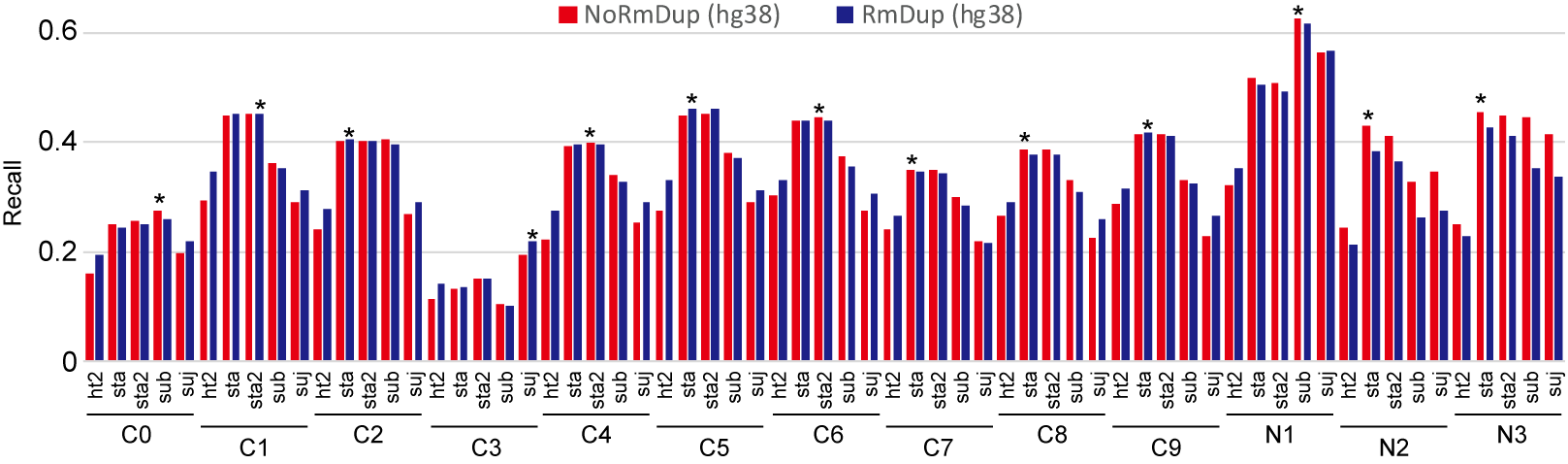

Supplement: S1 Fig — Recall was defined as the number of overlapped pRESs between merged RNA-seq and individual RNA-seq divided by the total number of pRESs identified in the given merged RNA-seq data (bottom). (TIF) [file pone.0201822.s001.tif]

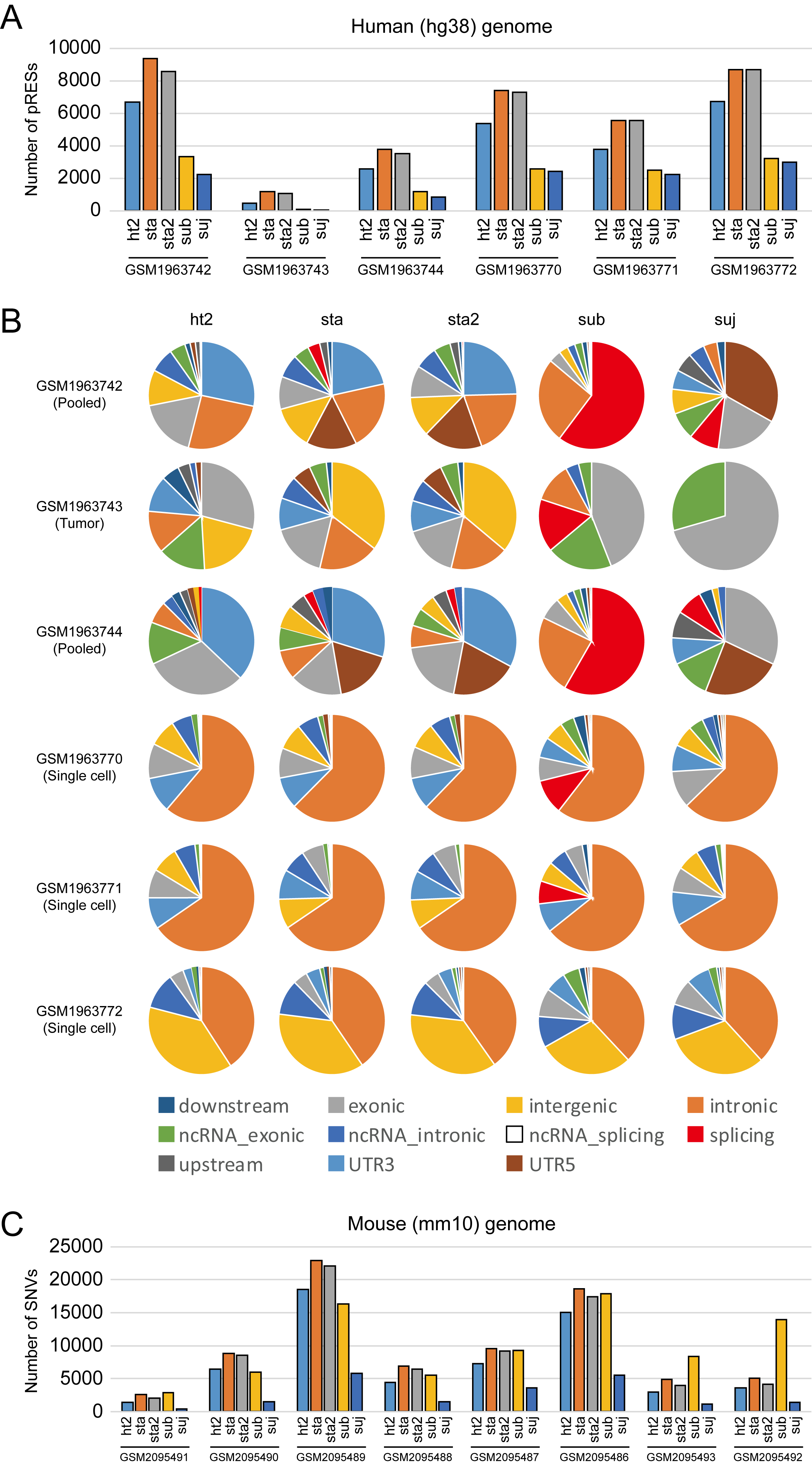

Supplement: S2 Fig — (A) The bar graph shows the number of pRESs identified in the six samples from a human RNA-seq data set (GSE75688) [28]. (B) Pie charts show the proportions of pRESs according to coding and noncoding (nc) gene-related features. (C) The bar graph shows the number of single-nucleotide variants (SNVs; not filtered by known single nucleotide polymorphisms) identified in the eight samples from a mouse RNA-seq data set (GSE79447). (TIF) [file pone.0201822.s002.tif]
